# Supplementary material for: Content-rich biological network constructed by mining PubMed abstracts
Source: BMC Bioinformatics. 2004 Oct 8;5:147. doi: 10.1186/1471-2105-5-147 (PMC528731; doi:10.1186/1471-2105-5-147)
Supplement: Additional File 2 — The original results of the above study (non-essential files are deleted to keep the file size under the limit set by BMC bioinformatics). [file 1471-2105-5-147-S2.bz2 › chilibotAdditionalFile2/dip30/102ID7937747E432/html/MCL1_BCL2.html]

 


 **MCL1** and **BCL2** 
  
Found 328 abstracts in PubMed, retrieved 30.  
 

 What does Google say? 
 PDF only 
| .edu only 

---

**Interactive relationship** (e.g. stimulation, inhibition, etc)

**Neutral relationship**- gossypol induced apoptosis triggers down regulation of anti apoptosis Bcl 2  [ **BCL2** ]  members Bcl X L, Bag 1 and Mcl 1  [ **MCL1** ] , up regulation of pro apoptosis Bcl 2  [ **BCL2** ]  member Bak, activation of caspase,, and 9, up regulation of Apaf 1, release of cytochrome c cyto c from mitochondria, and activation of both DFF45 and PARP.  Ref: 12818369 Biochem Pharmacol, 2003
- Here we show that Bid, a crucial proapoptotic protein that integrates the action of other Bcl 2  [ **BCL2** ]  family members, exhibits discrete specificity for metabolites of cardiolipin, especially monolysocardiolipin MCL  [ **MCL1** ] .  Ref: 12894218 Cell Death DifferCell Death Differ, 2003
- MCL  [ **MCL1** ]  may thus play a crucial role in connecting lipid metabolism, relocation of Bid to mitochondria and integrated action of Bcl 2  [ **BCL2** ]  proteins on mitochondrial membranes.  Ref: 12894218 Cell Death DifferCell Death Differ, 2003

**Non-interactive relationship** (e.g. studied together, co-existance, homology, etc.)

- Using immunohistochemical methods and a 10% cut off, each protein was detected in a subset of PTCLs BCL 2  [ **BCL2** ]  in 46%, BCL XS in 49%, BAX in 57%, BCL XL in 57%, and MCL 1  [ **MCL1** ]  in 65%.  Ref: 12754745 J Pathol, 2003
- Co administration of 17 AAG and UCN 01 did not modify expression of Hsp90, Hsp27, phospho JNK, or phospho p38 MAPK, but was associated with further p34 cdc2 dephosphorylation and diminshed expression of Bcl 2  [ **BCL2** ] , Mcl 1  [ **MCL1** ] , and XIAP.  Ref: 12738674 Blood< MedlineTA>Blood, 2003
- The present study investigated expression levels of the anti apoptotic proteins BCL 2  [ **BCL2** ] , BCL XL and MCL 1  [ **MCL1** ]  and the pro apoptotic proteins BAX and BCL XS in a series of 112 peripheral T cell lymphomas PTCLs classified according to the WHO classification.  Ref: 12754745 J Pathol, 2003
- The stress induced transcription factor GADD153 and the  **Bcl2**  related  [ **MCL1** ]  protein Bak are upregulated in response to fenretinide.  Ref: 12880976 Cancer Lett, 2003
- This study examined cutaneous T cell lymphoma CTCL cell lines and cutaneous lesions for the presence of Bcl 2  [ **BCL2** ]  gene family members and found that the two apoptosis inhibiting members Bcl xL and Mcl 1  [ **MCL1** ]  and the two apoptosis supporting members Bad and Bax were expressed.  Ref: 12754746 J Pathol, 2003
- The study aim was to investigate Stat 3 activation in a series of multiple myeloma MM cases and its effect on downstream targets such as the anti apoptotic proteins Bcl xL, Mcl 1  [ **MCL1** ] , and Bcl 2  [ **BCL2** ] , and the cell cycle protein cyclin D1.  Ref: 12707028 Am J Pathol, 2003
- Much less is known about the role the more recently described Bcl 2  [ **BCL2** ]  family member Mcl 1  [ **MCL1** ]  might play in tumor biology and resistance to chemotherapy.  Ref: 12787138 J Invest Dermatol< MedlineTA>J Invest Dermatol, 2003
- Out of the 10 Bcl 2  [ **BCL2** ]  family members, only the Mcl 1  [ **MCL1** ]  gene was regulated by IL 6.  Ref: 12771946 Oncogene, 2003
- It was revealed that LIGHT treatment resulted in down regulation of anti apoptosis Bcl 2  [ **BCL2** ]  family member Bcl 2  [ **BCL2** ] , Bcl X L, Bag 1, and Mcl 1  [ **MCL1** ] .  Ref: 12767529 Cancer Lett, 2003
- Immunohistochemistry was performed on paraffin sections using antibodies against cyclin D1, Bcl 2  [ **BCL2** ] , Bcl xL, Mcl 1  [ **MCL1** ] , p21, Stat 3, and Stat 3 phosphorylated P.  Ref: 12707028 Am J Pathol, 2003
- this is paralleled by higher Bcl 2  [ **BCL2** ] , Bcl xL, Mcl 1  [ **MCL1** ] , Pgp and lower Bax expression levels.  Ref: 12745269 Haematologica, 2003
- Expression of the anti apoptosis proteins, Bcl 2  [ **BCL2** ]  and Mcl 1  [ **MCL1** ]  expression decreased time dependently while Bax expression increased.  Ref: 12883267 Pancreas, 2003
- However, the expression levels of Bcl 2  [ **BCL2** ] , Bax, Bak and Mcl 1  [ **MCL1** ]  were unchanged.  Ref: 12842996 Blood< MedlineTA>Blood, 2003
- These inhibitors were found to be Mcl 1  [ **MCL1** ]  and Bcl xL, two antiapoptotic members of the Bcl 2  [ **BCL2** ]  family.  Ref: 12783855 Genes Dev< MedlineTA>Genes Dev, 2003
- EAT mcl 1  [ **MCL1** ]  EAT, a bcl 2  [ **BCL2** ]  related anti apoptotic gene, is up regulated at the early stage of differentiation of human embryonal carcinoma cells.  Ref: 12782407 Mol Cell Endocrinol, 2003
- The expression of Fas antigen, p21, p53, bax and  **bcl2**  related  [ **MCL1** ]  to radiation induced apoptosis was comparable to that of the caffeine untreated group.  Ref: 12792986 In Vivo< MedlineTA>In Vivo, 2003
- In contrast, treatment of cells with LY294002, to inhibit phosphoinositide 3 kinase PI3K, caused downregulation of Bcl 2  [ **BCL2** ]  and Mcl 1  [ **MCL1** ]  and allowed DeltaMEKK1 ER\* to elicit a robust apoptotic response characterized by activation of Bax and caspases.  Ref: 12879014 Oncogene, 2003
- In contrast to Bcl xL and Mcl 1  [ **MCL1** ] , the expression of Bcl 2  [ **BCL2** ]  showed an inverse correlation with proliferation rate P 0.0003.  Ref: 12707028 Am J Pathol, 2003
- Hypoxia induced cell cycle arrest at the G0 G1 phases and massive cell apoptosis after 24 hours through a reduction in the Bcl 2  [ **BCL2** ]  to Bax ratio, downregulation of Bcl XL and Mcl 1  [ **MCL1** ] , and upregulation of caspase 3 and caspase 8.  Ref: 12746487 J Cell Sci< MedlineTA>J Cell Sci, 2003
- Islet cell hyperplasia in transgenic mice overexpressing EAT mcl 1  [ **MCL1** ] , a bcl 2  [ **BCL2** ]  related gene.  Ref: 12782407 Mol Cell Endocrinol, 2003
- In the same way, BFA did not alter Bcl 2  [ **BCL2** ] , Bax and Mcl 1  [ **MCL1** ]  expression.  Ref: 12759546 Oncology, 2003
- Activation of these pathways contributes not only to the expression of genes that cause inflammation and destruction but also to the expression of a variety of anti apoptotic molecules, including FLICE inhibitory protein, Bcl 2  [ **BCL2** ] , and Mcl 1  [ **MCL1** ] , which protect against apoptosis that may be initiated through death receptor or mitochondria dependent pathways.  Ref: 12810199 Curr Opin Pharmacol, 2003
- Antisense therapy against the Mcl 1  [ **MCL1** ]  gene product, possibly in combination with antisense strategies targeting other antiapoptotic Bcl 2  [ **BCL2** ]  family members, appears to be a rational and promising approach to help overcome treatment resistance of malignant melanoma.  Ref: 12787138 J Invest Dermatol< MedlineTA>J Invest Dermatol, 2003
- inhibition of TNF alpha induced activation and expression of phosphatidylinositol 3 kinase PI3K Akt, and also blockage of expression of Mcl 1  [ **MCL1** ] , an antiapoptotic Bcl 2  [ **BCL2** ]  family member which is located downstream of Akt, was inhibited by IEX 1S.  Ref: 12682234 J Immunol, 2003
- The dramatic potentiation of CDK inhibitor induced apoptosis by LY was accompanied by diminished Bad phosphorylation, induction of Bcl 2  [ **BCL2** ]  cleavage, and down regulation of X linked IAP and Mcl 1  [ **MCL1** ] .  Ref: 12702569 Cancer Res, 2003
- All multidomain anti apoptotic members Bcl x, Bcl 2  [ **BCL2** ] , Mcl 1  [ **MCL1** ] , Bcl w and Bcl2l10 Diva Boo except Bcl2a1 A1 are expressed in OLC.  Ref: 12787069 J Neurochem, 2003
- all were BCL 2  [ **BCL2** ]  negative and MCL 1  [ **MCL1** ]  positive.  Ref: 12754745 J Pathol, 2003
- Remarkably, these events occurred in the absence of any reduction in the expression of the Bcl 2  [ **BCL2** ]  family members Bcl 2  [ **BCL2** ] , Mcl 1  [ **MCL1** ]  and Bcl XL, or any change in the proapoptotic molecules Bad or Bax.  Ref: 12842827 Am J Physiol Gastrointest Liver Physiol< MedlineTA>Am J Physiol Gastrointest Liver Physiol, 2003
- The proliferation index, assessed by the MIB 1 antibody, correlated with expression levels of MCL 1  [ **MCL1** ]  R = 0.42, p = 0.003, BCL 2  [ **BCL2** ]  R = 0.32, p = 0.027, BAX R = 0.33, p = 0.014, and BCL XL R = 0.34, p = 0.015 Spearman rank.  Ref: 12754745 J Pathol, 2003
- The expression of Bcl 2  [ **BCL2** ] , Bcl xL, and Mcl 1  [ **MCL1** ]  may therefore be sufficient to guarantee the survival of malignant CTCL cells.  Ref: 12754746 J Pathol, 2003
